# Specialized Pro-Resolving Lipid Mediators in Pulmonary Diseases: Molecular and Therapeutic Implications

**DOI:** 10.3390/molecules30102212

**Published:** 2025-05-19

**Authors:** Ángel Ortega, Pablo Duran, Bermary Garrido, Alexander Manzano, Carolina Navarro, Aljadis Silva, Milagros Rojas, Juan Bautista De Sanctis, Danuta Radzioch, Diego Rivera-Porras, Carlos Silva Paredes, Valmore Bermúdez

**Affiliations:** 1 Endocrine and Metabolic Diseases Research Center, School of Medicine, University of Zulia, Maracaibo 4001, Venezuela; angelort94@hotmail.com (Á.O.); pabloduran1998@gmail.com (P.D.); bermarygarrido@gmail.com (B.G.); amanzano_8@hotmail.com (A.M.); cnm10698@gmail.com (C.N.); aljadisenrique@gmail.com (A.S.); migarocafi@gmail.com (M.R.); 2 Institute of Molecular and Translational Medicine, Faculty of Medicine and Dentistry, Palacký University Olomouc, 77900 Olomouc, Czech Republic; juanbautista.desanctis@upol.cz; 3 The Research Institute of the McGill, University Health Center, McGill University, Montreal, QC H0H H9Z, Canada; danuta.radzioch@mcgill.ca; 4 Universidad de la Costa, Departamento de Productividad e Innovación, Barranquilla 080001, Atlántico, Colombia; drivera23@cuc.edu.co; 5 Universidad del Zulia, Facultad de Medicina, Departamento de Ciencias Fisiológicas, Maracaibo 4001, Venezuela; csilva_paredes@hotmail.com; 6 Universidad Simón Bolívar, Facultad de Ciencias de la Salud, Centro de Investigaciones en Ciencias de la Vida, Barranquilla 080001, Atlántico, Colombia

**Keywords:** pulmonary diseases, PUFAs, specialized pro-resolving lipid mediators, inflammation, COPD

## Abstract

Inflammatory lung diseases (ILDs) represent a global public health crisis characterized by escalating prevalence, significant morbidity, and substantial mortality. In response to the complex immunopathogenic mechanisms driving these conditions, novel pharmacological strategies targeting resolution pathways have emerged throughout the discovery of specialized pro-resolving lipid mediator (SPM; resolvins, maresins, and protectins) dysregulation across the ILD spectra, positioning these endogenous molecules as promising therapeutic candidates for modulating maladaptive inflammation and promoting tissue repair. Over the past decade, this paradigm has catalyzed extensive translational research into SPM-based interventions as precision therapeutics for respiratory inflammation. In asthma, they reduce mucus hypersecretion, bronchial hyperreactivity, and airway inflammation, with prenatal SPM exposure potentially lowering offspring disease risk. In COPD, SPMs attenuate amyloid A-driven inflammation, normalizing cytokine/chemokine imbalances and oxidative stress and mitigating COVID-19-associated cytokine storm, enhancing survival. This review synthesizes SPMs’ pharmacotherapeutic mechanisms in ILDs and evaluates current preclinical and clinical evidence.

## 1. Introduction

Inflammatory lung diseases (ILDs) like asthma, chronic obstructive pulmonary disease (COPD), cystic fibrosis (CF), and COVID-19-related pulmonary sequelae impose significant global health burdens through both morbidity and mortality. Asthma remains the most prevalent ILD, affecting ~235 million people worldwide and serving as the paradigm of chronic airway inflammation [1]. COPD follows as the most lethal, accounting for 3.5 million deaths in 2021 [2]; meanwhile, CF is the predominant autosomal recessive disorder in Caucasian populations [3], while COVID-19 has caused 777 million confirmed cases and 7.1 million deaths globally since its emergence in December 2019 to February 2025 [4].

Current management of inflammatory lung diseases (ILDs) remains palliative, focusing on symptom mitigation rather than curative intervention. The growing epidemiological burden of ILDs, combined with the lack of disease-modifying therapies and the limitations of conventional corticosteroids (including immunosuppression, treatment resistance, and exacerbation risk), creates clinical and economic challenges for the healthcare system worldwide [5,6,7,8]. Consequently, extensive research efforts over the past decade have sought to identify novel therapeutic targets capable of durably resolving rather than temporarily suppressing the dysregulated inflammatory responses characteristic of these diseases.

This paradigm shift aims to achieve more sustained improvements in pulmonary function while minimizing treatment-related complications [9], in which the main therapeutic target is the immune system and the native cells of the respiratory system, fighting against the dysfunction caused either by genetic factors (e.g., mutation in the CFTR gene) and/or foreign substances such as smoke from cigarettes, certain allergens, viruses, and other chemical environmental substances, which leads to morpho-functional alterations of the airways, manifesting as clinical signs and symptoms of the aforementioned entities [10,11,12].

Among the new therapeutic options for ILD management, specialized pro-resolving lipid mediators (SPMs), small molecules derived from polyunsaturated fatty acids (PUFAs), omega-6 (ω-6), and omega-3 (ω-3) [13,14,15,16], have attracted great interest in recent years. SPMs exhibit anti-inflammatory and pro-resolution properties related to the modulation of cells involved in the pathophysiology of ILD, as well as in lung tissue repair and lower bronchial hyperreactivity [9,17,18].

In this context, this review aims to articulate the pharmacotherapeutic mechanisms through which SPMs act in lung diseases and summarize the current preclinical and clinical evidence regarding the impact of SPMs in their treatment.

## 2. Materials and Methods

This review provides an information synthesis on the function of specialized pro-resolving lipid mediators in pulmonary diseases through a non-systematic extensive literature search on Scopus, EMBASE, PubMed, ISI Web of Science, ScienceDirect, Medline, Cochrane Library Plus, and Google Scholar databases from inception to March 2025. Only articles in Spanish and English were used. No restrictions were made based on the type of study. Scientific articles from high-impact journals were selected: Q1, Q2, and Q3. The studies used for the preclinical and clinical evidence section were selected, for the most part, based on the clarity and reproducibility of their methodology and the fact that they were of high scientific quality. The search strategy included terms such as “pulmonary diseases”, “PUFAs”, “specialized pro-resolving lipid mediators”, and “inflammation” combined with Boolean operators (AND/OR).

## 3. Results and Discussion

### 3.1. Specialized Pro-Resolving Lipid Mediators: An Overview

**Figure 1:**
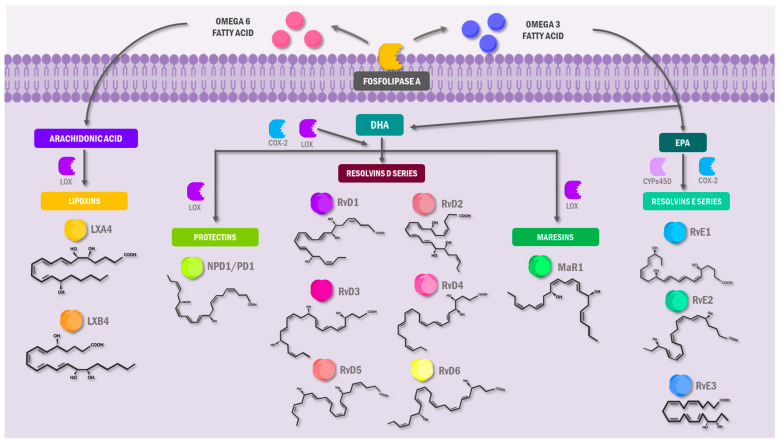
**Metabolism of specialized pro-resolving lipid mediators.** Distribution of different enzymes, intermediaries, and precursors that actively participate in the biosynthesis of SPM during the last stages of inflammation, which develop anti-inflammatory agents that limit tissue damage, PMN infiltration, and elimination of causal agents. ω-3 and ω-6 PUFAs are the starting point for elaborating these pro-resolution molecules. SPM: specialized lipid mediator; PMN: polymorphonuclear cells; ω-3; ω-6; EPA: eicosapentaenoic acid; DHA: docosahexaenoic acid; RvE1: resolvin E1; RvE2: resolvin E2; RvE3: resolvin E3; RvD1: resolvin D1; RvD2: resolvin D2; RvD3: resolvin D3; RvD4: resolvin D4; RvD5: resolvin D5; RvD6: resolvin D6; NPD1: neuroprotectin 1; MaR1: maresin 1; LXA4: lipoxin A4; LXB4: lipoxin B4; LOX: lipoxygenase; COX-2: cyclooxygenase 2; CYPs: cytochromes P450.

SPMs are a large family of endogenous lipids that limit physiological pro-inflammatory responses [19]. SPMs can exhaustively regulate inflammation and limit tissue damage by self-limiting the infiltration of neutrophils, promoting their apoptosis, and inhibiting the proliferation of pro-inflammatory substances in the lesion site [20]. These lipids are biosynthesized from ω-6 and ω-3 polyunsaturated fatty acid (PUFA) precursors, with arachidonic acid (AA; 20:4ω-6) constituting the major ω-6 metabolite and eicosapentaenoic acid (EPA; 20:5ω-3), docosahexaenoic acid (DHA; 22:6ω-3), and docosapentaenoic acid (DPA; 22:5ω-3) comprising the principal ω-3 derivatives (Figure 1) [21,22]. SPMs appear chronologically due to enzymatic reactions involving lipoxygenases (LOXs), enabling more efficient resolution of inflammation. These SPMs include lipoxins (LXs), which derive from AA, and maresins (MaRs), protectins (PDs), and resolvins (Rvs), which derive from ω-3 [23].

Likewise, each SPM has individual derivates, allowing for classification based on the fatty acid required for its synthesis. For example, LXs are derived from AA, an important precursor in pro-inflammatory and anti-inflammatory molecules [24]. Lipoxin (LX) biosynthesis involves sequential oxygenation of arachidonic acid (AA) by 15-lipoxygenase (15-LOX) and 5-lipoxygenase (5-LOX), generating two stereospecific isoforms: lipoxin A_4_ (LXA_4_) and lipoxin B_4_ (LXB_4_). These bioactive metabolites are endogenously produced in human vasculature, platelets, and lymphocytes, functioning as potent resolution-phase mediators [25].

While lipoxins (LXs) are endogenously biosynthesized, exogenous compounds like aspirin stimulate an alternative pathway by cyclooxygenase (COX) acetylation, modifying its enzymatic activity to generate 15R hydroperoxy eicosatetraenoic acid (15R HpETE). This intermediate is the 5-lipoxygenase (5 LOX) substrate, enhancing the production of 15 epi lipoxin A4 (15 epi LXA4) [26]. Furthermore, low-dose aspirin does not interfere with the active synthesis of prostacyclin (PG12), and both mediators, LXA4 and PG12, are considered anti-inflammatory substances with an important role in the resolutive phases [27].

Resolvins consist of two series: The E-series (RvE) is derived from EPA, and the D-series (RvD) is derived from DHA. The E-series results in RvE1 through the action of numerous enzymatic reactions that mainly involve 5-LOX [28]. Likewise, RvD is enzymatically derived from DHA, a substrate for the 15-LOX and 5-LOX enzymes, to form 17S-hydroperoxy-DHA (17S-H (p) DHA). This can result in RvD1, RvD2, RvD3, and RvD4 [29]. Interestingly, ω-3 fatty acids and aspirin work together against inflammation, as with LXs. Aspirin can trigger Rvs synthesis in two series. Meanwhile, DHA converts to 17R-hydroxy-DHA through oxygenation pathways initiated by COX-2. The result is aspirin-triggered RvD (AT-RvD), a pathway similar to the one needed to obtain RvE [30].

DHA is a precursor for lipid mediators, such as PDs and MaRs, using the lipoxygenation pathways [31]. PD 1, which is derived from DHA, can also be identified as neuroprotectin 1 (NPD) when acting on the neural tissue [32]. Conversely, macrophages synthesize MaRs through the initial lipoxygenation of the carbon at the 14th position, producing 13S- and 14S-epoxide-maresin and further enzymatically converting into members of the MaRs family: MaRs 1, MaRs 2, and MaRs conjugate in tissue regeneration (MCTR) [33,34].

### 3.2. Pro-Resolving Lipid Mediators in Pulmonary Diseases: Molecular Mechanisms

Recently, SPMs have emerged as a potentially effective therapeutic target for ILD due to their pro-inflammatory and pro-resolutive properties. In the following sections, we discuss the possible pharmacotherapeutic mechanisms of SPMs in different ILDs. Although most of the preclinical evidence has described SPMs’ role in ILD, it is important to note that authors such as Mas et al. [35] have demonstrated some SPMs, such as RvD1 and RvD2, in blood after some weeks of ω-3 supplementation, supporting the potential efficacy of SPM mechanisms in ILD described below and supported in the clinical evidence section later in the paper.

#### 3.2.1. Asthma

Asthma is a common inflammatory disorder characterized by bronchial hyperactivity resulting in bronchial smooth muscle contraction and airway remodeling, edema, subepithelial fibrosis, and mucous hypersecretion, leading to respiratory distress and limited airflow [36]. Asthma is characterized by hyperresponsiveness to environmental triggers, including allergens, pollutants, and viral pathogens. The underlying type 2 immune response involves coordinated activation of mast cells, dendritic cells, and Th2 lymphocytes within the bronchial mucosa. These cellular components drive pathology through the release of canonical type 2 cytokines (IL-5, IL-13), eicosanoids (prostaglandins, leukotrienes), and recruitment of eosinophils—the hallmark inflammatory infiltrate in asthmatic airways [36].

An important role for type 2 innate response lymphoid cells (ILC2s) has also been reported. These are instrumental in the response to allergic asthma, and they are activated by cytokines derived from epithelial cells and prostaglandin D2 (PGD2) derived from mastocytes. They can induce bronchial hyperactivity independently from adaptive immunity by producing IL-13 and eosinophilia through IL-5 production [37]. The persistence of all these mechanisms with the subsequent lack of resolution of chronic inflammation could lead to the continuity of this pathology [9,38]. These mediators have been widely recognized for their pro-resolution functions in the inflammation present in different pathologies [16,20,31]. In this regard, a study by Townsend et al. [39] showed how modulation of macrophages from asthma patients can be associated with the synthesis of SPMs such as LXA4 and RvD1. Thus, SPMs have been considered as a possible therapeutic target in asthmatic patients to resolve the chronic inflammation that characterizes the disease.

**Figure 2:**
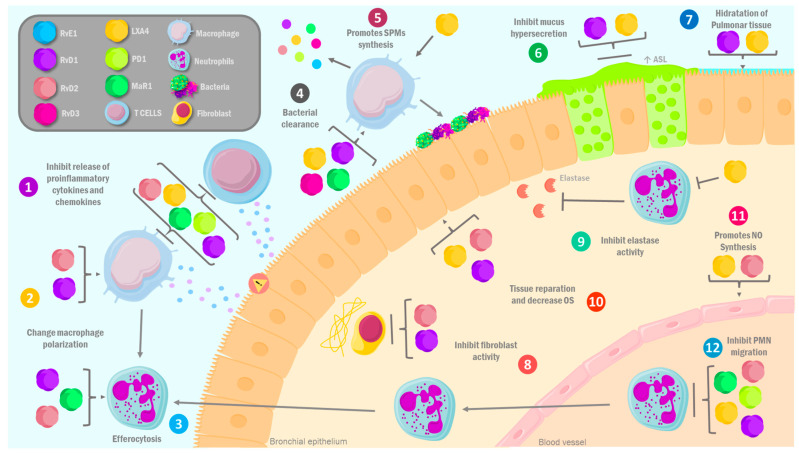
**Therapeutic mechanisms of specialized pro-resolving lipid mediators in chronic inflammatory lung diseases.** SPMs can function as therapeutic agents in various ILDs, such as asthma, COPD, CF, and COVID-19, by intervening in their pathophysiology through various anti-inflammatory and pro-resolution mechanisms: (1) inhibition of the release of pro-inflammatory cytokines and chemokines, (2) changes in the polarization of macrophages from pro-inflammatory phenotype M1 to anti-inflammatory M2, (3) promotion of macrophage-mediated spherocytosis of PMNs, (4) stimulation of phagocytosis of foreign bodies and bacteria, (5) increased synthesis of SPMs by macrophages, (6) inhibition of the activity of mucus-producing glands, (7) increased hydration of pulmonary epithelial tissue, (8) inhibition of fibroblast activity, (9) inhibition of elastase released by neutrophils, (10) regeneration and compensation of pulmonary epithelium, accompanied by a decrease in the apoptosis of lung epithelial cells and a decrease in oxidative stress, (11) promotion of nitric oxide synthesis, and (12) inhibition of transepithelial and transendothelial migration of PMNs. SPMs: specialized lipid mediators; PMN: polymorphonuclear cells; RvE1: resolvin E1; RvD1: resolvin D1; RvD2: resolvin D2; RvD3: resolvin D3; PD1: protectin 1; MaR1: maresin 1; LXA4: lipoxin A4; OS: oxidative stress; NO: nitric oxide; ASL: mucociliary clearing; ILD: inflammatory lung disease; COPD: chronic obstructive pulmonary disease; CF: cystic fibrosis.

The receptors of the SPMs, known to be G protein-coupled receptors, are present in the respiratory tract. Specifically, ALX/FPR2, which is a ligand of LXA4 and RvD1, is present in the epithelium of the respiratory tract, as well as in macrophages, eosinophils, T cells, ILC2, and monocytes [40]. In this context, asthma has been associated with decreased LX production [41], specifically in the case of LXA, which has been studied as an endogenous mediator of mucosal inflammation, reducing the severity of allergic and asthmatic reactions (Figure 2) [42]. It exerts this function by coupling with ALX/FPR2, and in the case of LXA4, binding to ALX/FPR2 results in changes at the cellular cytoskeleton’s phosphorylation level. In macrophages, for example, it generates activation of small GTPases, which induce the change in cytoskeletal proteins, whereas in neutrophils, these changes are triggered by the ability to inhibit the phosphorylation of protein kinase C-BII (PKCBII). This step leads to the activation of polyisoprene diphosphate phosphatase 1 (PDP1), a phosphatase that converts polyisoprenyl phosphates present in the cell membrane to pre-squalene diphosphate (PSDP) in its pre-squalene monophosphate (PSMP) form. The latter is a facilitator of functional cell responses involved in the restriction of pro-inflammatory cellular responses [20,43], such as chemotaxis inhibition; inhibition of migration and activation of neutrophils; promotion of apoptotic neutrophil phagocytosis by macrophages, eosinophilic migration, and apoptosis inhibition in inflammation areas; and the regulation of pro-inflammatory cytokine release by epithelial cells [44,45].

ALX/FPR2 receptor is also expressed by natural killer (NK) cells in asthma, which induces eosinophil apoptosis after LXA4 activation, regulating eosinophilia and inhibiting NK cell cytotoxicity. Furthermore, LXA4 blocks histamine release by mast cells during their interaction with bronchial epithelial cells, whereas LXB4 decreases its receptor expression, affecting IL-13 interactions between IL13 and IL-13Ra and IL-13Ra2. Similarly, LXB4 inhibits Th2 cell activation, cytokine release, and neutrophil chemotaxis [46]. Both LXA4 and LXB4 inhibit leukotriene B4-induced neutrophil chemotaxis (LTB4), and they inhibit chemotaxis induction and the degranulation of eosinophils by the platelet-activating factor (PAF), functions performed by competition with these receptor-related molecules [47]. LXA4 also has anti-inflammatory properties through the partial antagonism of other targets such as CysLT1 and leukotriene D4 receptors (LTD4) expressed in the lung tissue, causing bronchoconstriction when the aforementioned receptors become activated [46,48]. Similarly, through molecular competition, they can attenuate leukotriene C4 (LTC4)-induced bronchoconstriction [49].

Of the E-series Rvs, the antagonism of RvE1 to BLT1 is particularly relevant. BLT1 is a receptor with LTB4 affinity, acting as its counter-regulator. LTB4 is a chemotaxis, plasma exudation inducer, and lung parenchyma reducer [19,50]. RvE1 performs a function similar to that of LXA4 in NK cells, expressing the ChemR23/ERV1 receptor, which is a ligand for RvE1. When sensitized, the receptor promotes eosinophil apoptosis [51] through its capacity to regulate PI3K/AKT signaling, leading to the consequent activation of proteins involved in phagocytosis [52]. Additionally, in murine models, it was determined that RvE1 is one of the main inhibitors of Th2 cytokine production (IL-5, IL-3), which is widely involved in asthma [53]. It also decreases the production of pro-inflammatory Th17 cytokines, including IL-6 and IL-13, produced by macrophages, and IL-23, produced by lymphocytes. Similarly, it increases the production of LXA4 and IFN-γ. These cellular events have been associated with resolving allergic inflammation and mucus hyperproduction [9].

RvD1 and AT-RvD1 exhibit some measurable ALX/FPR2 receptor affinity and, thus, can initiate the pro-resolution signaling pathways at high concentrations. However, their binding to ALX/FPR2 is competitively inhibited by LXA4, demonstrating a higher receptor affinity and exhibiting receptor activation at significantly lower concentrations [54]. RvD1 also binds to the GPR32/DRV1 receptor with a shorter plasma half-life than ALX/FPR2. Expressed predominantly on macrophages, monocytes, and polymorphonuclear cells (PMNs), GPR32/DRV1 is activated by nanomolar concentrations of RvD1, enabling precise spatial and temporal regulation of resolution responses. Notably, RvD1’s primary pro-resolution effects require coordinated signaling through both receptor systems, with ALX/FPR2 interaction being particularly crucial for its anti-inflammatory functions. However, when RvD1 additionally sensitizes GPR32, this effect is even more pronounced [31]. A key mechanism of RvD1 action occurs through microRNA (miRNA) induction, which regulates critical inflammatory pathways. Specifically, RvD1 induces miR-21 to enhance IL-10 expression, promotes miR-146b to modulate the TNF-α–nuclear factor kappa B (NF-κB) axis (a central inflammatory and apoptotic pathway), and upregulates miR-219 to control CD14 and 5-LOX expression. This latter regulation is particularly significant, as 5-LOX is the key enzyme responsible for producing asthma-related leukotrienes such as LTB4, LTC4, LTD4, and SPM [52].

RvD1 has two main functions during the inflammation resolution: (1) activation of eosinophil apoptosis and (2) alveolar macrophage activation inducing neutrophil apoptosis in the airway. Similarly, it is important in the regulation of the TNF-α–NF-κB, which has a role in numerous areas of asthma pathology, acting as a chemoattractant for neutrophils and eosinophils and an inducer of the excessive airway response seen in asthma [51,55]. In addition, RvD1 inhibits the change of IgG to IgE, stabilizing the regulatory protein BCL-6 that prevents the coupling of the transcription factor STAT6 to the e-germline transcript (eGLT) promoter. This results in decreased expression of eGLT, a key factor regulating the processing of IgG to IgE, the predominant immunoglobulins in allergic diseases like allergic asthma [53,56].

Similarly, MaRs orchestrate inflammation resolution by driving macrophage sferocytosis of apoptotic neutrophils (e.g., in the lungs) and polarizing M1 macrophages toward an M2 anti-inflammatory phenotype [34,57,58]. MaR1 serves as a key mediator of the ILC2 regulatory pathway. MaR1 suppresses Th2 cytokine production in murine models while upregulating amphiregulin expression—a mucosa-protective factor secreted by ILC2s. Additionally, MaR1 enhances the expression of Forkhead box P3 (Foxp3), a critical transcription factor in regulatory T (Treg) cells, which promotes ILC2-Treg crosstalk and attenuates Th2-mediated allergic asthmatic responses. [59].

Emerging evidence suggests that PD1 is a critical regulator of allergic inflammation resolution in asthma, with PD production occurring systemically (Th2 CD4+ lymphocytes) and locally (respiratory epithelium-resident eosinophils and leukocytes). [60]. Although its receptor remains unidentified, this mediator appears to function through NF-κB pathway inhibition, leading to reduced COX-2 expression and decreased prostaglandin (particularly PGD2) production—a key mechanism in airway hyperreactivity [46]. Additionally, PD1 attenuates 15-LOX expression, thereby limiting leukotriene biosynthesis [32]. PD1 can also decrease allergic inflammation, lowering the trafficking of eosinophils and T lymphocytes, decreasing mucus production, and decreasing leukocyte infiltration, a function shared with RvD1 [61].

#### 3.2.2. Chronic Obstructive Pulmonary Disease (COPD)

Chronic obstructive pulmonary disease (COPD) ranks among the most prevalent and deadliest chronic respiratory conditions worldwide [62,63]. COPD is characterized by persistent, progressive, and irreversible airflow limitation [64] due to structural changes in both the airways and the alveoli due to chronic inflammation. These pathological alterations lead to characteristic clinical manifestations, including chronic cough (with or without sputum production) and exertional dyspnea [65,66]. While genetic factors (such as α-1 antitrypsin deficiency) may contribute, cigarette smoke exposure remains the primary etiological factor. Tobacco smoke induces a chronic pro-inflammatory state that promotes parenchymal destruction (emphysema) and small airway remodeling—the hallmark pathological features of COPD, alongside other factors like oxidative stress, pro-inflammatory chemokines and cytokines, macrophages, neutrophils, and T and B lymphocyte infiltration [67,68,69].

The search for novel therapeutic agents for COPD management has intensified in recent years, driven by the significant adverse effects of current treatments [70,71]. Like other chronic inflammatory lung diseases, COPD exhibits reduced SPM levels [72], highlighting its critical role in disease pathogenesis. These endogenous mediators have emerged as promising therapeutic candidates by their ability to (1) activate pro-resolution mechanisms, (2) prevent and modulate excessive inflammation, and (3) protect against progressive lung tissue remodeling [73].

Lipoxin A_4_ (LXA_4_) and aspirin-triggered lipoxins (ATLs)—key specialized pro-resolving mediators (SPMs) in COPD—act as ALX/FPR2 receptor-negative allosteric modulators. Through this mechanism, they antagonize the pro-inflammatory effects of serum amyloid A (SAA), which is elevated during COPD exacerbations and drives corticosteroid-resistant inflammation. SAA has pro-inflammatory properties, including the induction of IL-8 production, the activation of neutrophil elastase, and its recruitment in the pulmonary tissue [52,73,74,75,76]. Beyond receptor modulation, LXA_4_ and ATL exert multifaceted protective effects by (1) enhancing ALX/FPR2-dependent macrophage efferocytosis, (2) directly interacting with airway epithelial cells to inhibit metalloproteinases and pro-inflammatory mediators (IL-1β, IL-17, TNF-α), and (3) reducing leukocyte transendothelial migration and collectively promoting tissue repair while dampening chronic inflammation [77,78].

Alternatively, Rvs binding to ALX/FPR2 and GPR32 in macrophages, neutrophils, and mucosal pulmonary cells plays a crucial role in inflammation caused by cigarette smoke [79]. In addition, Rvs decrease cigarette oxidative stress by suppressing nitrosylation markers like 8-OHdG and nitrotyrosine, inhibiting H_2_O_2_ production in bronchial epithelial cells [80,81]. Furthermore, AT-RvD1 and RvD1 demonstrate robust therapeutic efficacy in emphysema models, markedly suppressing cigarette smoke-induced alveolar space enlargement through their pro-resolving mechanisms. These specialized pro-resolving mediators exert their protective actions through (1) inhibition of smoke-induced pulmonary cell apoptosis, (2) reduction of inflammatory cell infiltration, (3) suppression of goblet cell hyperplasia and mucus hypersecretion, and (4) promotion of tissue repair mechanisms. These multimodal effects likely stem from their ability to modulate immune cell function and resident lung cell populations, including alveolar epithelial cells and fibroblasts [12,80].

Rvs suppress the production of pro-inflammatory chemokines and cytokines such as MCP-1, PGE_2_, IL-6, IL-8, and COX-2 by bronchial fibroblasts [73]. Specifically, RvD1, RvD2, and MaR1 influence other cells involved in the development of the disease, such as T lymphocytes, by decreasing the synthesis of pro-inflammatory cytokines by lymphocytes T CD8+, T CD4+, Th1, and Th17. Likewise, they downregulate specific transcription genes such as t-bet and Rorc, which permit the differentiation of CD4+ to Th1 and Th17 [82]. Likewise, RvD1, one of its derivatives (17R-RvD1), and RvD2 can modulate the activity of macrophages and neutrophils, two key components in COPD pathophysiology [79].

Rvs drive a profound phenotypic switch in pulmonary macrophages, shifting their polarization from pro-inflammatory M1 to anti-inflammatory M2 states. This reprogramming is characterized by a marked downregulation of M1-associated markers (iNOS, TNF-α) and pro-inflammatory cytokines (IL-6, IL-8, TNF-α), as well as M2 upregulation of signature molecules, including arginase-1 (Arg-1), its transcriptional regulators, mannose receptor 1 (Mrc1/CD206), and IL-10 [73], and suppression of COX-2-mediated inflammatory pathways, leading to reduced production of prostaglandins (PGD_2_, PGE_2_). Concurrently, Rvs enhance the secretion of reparative cytokines (IL-10, TGF-β), with TGF-β playing a pivotal role in alveolar repair and parenchymal restoration [12,79,83,84]. Furthermore, Rvs enhance macrophage-mediated bacterial phagocytosis in COPD and efferocytosis of apoptotic neutrophils, thereby resolving inflammation and promoting tissue repair [79]. These actions are modulated through signaling pathways such as the NF-κB, which RvD2 can inhibit by preventing p65, Ib, and p52 phosphorylation. Furthermore, an alternate pathway of NF-κB related to increased RelB presence also occurs. RelB is an important anti-inflammatory factor that could be related to the therapeutic properties of Rvs in COPD [79,85].

Maresin 1 (MaR1) modulates airway inflammation through targeted inhibition of protein kinase C (PKC) isoforms α and ε. Therefore, it is the most powerful pro-resolution mediator in spherocytosis induction and tissue repair and could be useful in helping patients with COPD [86]. This action leads to (i) downregulation of intercellular adhesion molecule 1 (ICAM 1) expression, (ii) attenuation of neutrophil infiltration, (iii) suppression of pro-inflammatory cytokine release (IL 6, TNF α), and (iv) inhibition of T lymphocyte activation and associated pro-inflammatory responses [34,59]. Notably, these immunomodulatory mechanisms parallel those described for protectin D1 (PD1) in similar pathological contexts [9].

#### 3.2.3. Cystic Fibrosis

Cystic fibrosis (CF) is one of the most prevalent inherited disorders worldwide [87]. This autosomal recessive condition stems from mutations in the cystic fibrosis transmembrane conductance regulator (CFTR) gene [88], manifesting as multi-organ pathology affecting the pancreas, liver, exocrine glands, reproductive tract, and pulmonary system [89]. The respiratory complications are particularly severe, as CFTR encodes a cAMP-regulated chloride channel (Cl^−^) critical for maintaining airway surface liquid homeostasis. Impaired Cl^−^ efflux disrupts mucociliary clearance through defective water transport across the pulmonary epithelium [90]. As expected, the mutation of this gene generates defective transports that lead to the accumulation of mucus on the surface, leading to acute unresolved pro-inflammatory environments and repeated viral and bacterial infections that destroy airways throughout the years [90].

Cystic fibrosis (CF) is a progressive and fatal pulmonary disorder characterized by mortality rates exceeding 90% in untreated cases, demanding comprehensive therapeutic approaches [90]. The disease pathophysiology establishes a self-perpetuating cycle of chronic inflammation marked by persistent neutrophil infiltration, dysregulated cytokine production, and impaired microbial clearance. Within this pathological context, specialized pro-resolving mediators (SPMs) have emerged as promising therapeutic candidates through their demonstrated ability to attenuate excessive neutrophilic recruitment, rebalance inflammatory mediator homeostasis, enhance pathogen clearance, and promote tissue repair mechanisms [91]. Importantly, a quick and effective resolution is crucial in pro-inflammatory environments; however, it has been observed that alterations in the metabolism of PUFAs lead to unbalanced levels of resolving mediators [92]. Studies have shown that there is a relation between CFTR-deficient cells and low levels of SPMs due to the abnormal activity of lipoxygenases; however, the exact pathway through which CFTR intervenes in the reduced expression of 5LO, 12LO, 15LO, and LTA4H has not been identified [93].

Nevertheless, SPMs such as LXA4 and RvD1 have been strongly linked to restoring pulmonary epithelium hydration and mucociliary clearance. These SPMs regulate ion transport independently of the CFTR protein, stimulating CL^−^ secretion and inhibiting the absorption of amiloride-sensitive Na^+^ [94,95]. Furthermore, LXA4 is involved in the apical secretion of adenosine triphosphate (ATP) through pannexin channels, activating purine receptors and triggering intracellular calcium signals [Ca (2^+^)] (i) in human bronchial epithelial cells. This mechanism needs the specific activation of PKC mediated by the stimulation of the ALX/FPR2 receptor in monocytes, neutrophils, and the pulmonary epithelium, as is the case of RvD1 [94,96]. Therefore, the increase in [Ca (2^+^)] (i) induced by LXA4 can play a key role not only as pro-resolution molecules but also in the autocrine production of LXA4, amplifying its endogenous biosynthesis [97].

Interestingly, airway dehydration is not the only problem in CF, as repeated lung infections, mainly by bacterial and fungal agents such as *Pseudomonas aeruginosa*, *Staphylococcus aureus*, *Mycobacterium abscessus*, and *Aspergillus fumigatus*, contribute to progressive deterioration of the lung function in these patients [98]. However, some SPMs, such as LXA4 and RvD1, induce limited production of pro-inflammatory substances such as IL-8, IL-1β, and IL-17. There is also decreased bacterial load, PMN infiltration, tissue damage, and a similarity in treating these patients with ciprofloxacin [99]. Karp et al. found that using LXA4 analogues delays bacterial colonization by improving transepithelial electric resistance and the expression of closed-junction protein ZO-1 in the plasma membrane of human bronchial epithelial cells. Also, the constant stimulation of LXA4 on ATP-sensitive potassium channels and the mitogen-activated protein kinase pathway promote tissue repair [100]. RvD1 also shows its activity in lung macrophages, where they regulate the expression of Toll-like receptors and subsequent genes and microRNAs (miR)-21 and -155, which leads to reduced inflammatory signaling. Furthermore, it has been described that LXA4 analogues manage to decrease the active secretion of IL-8 induced by surface-expressed TNF-α, improving phagocytic destruction capacity by protecting against tissue injury [10].

LXA4 also plays a leading role in the vascular endothelium, specifically in regulating inflammatory processes. RvD1, RvD2, and LXA4 reduce IL-1β-induced vascular permeability, limiting PMN adhesion and diapedesis. LXA4 and RvD2 stimulate NO production, which counteracts interactions between leukocytes, while SPMs like LXA4 and LXB4 limit the migration of PMN induced by leukotriene B4 (LTB4) [101]. In particular, it has been shown that incubation of macrophages with MaR1 triggers biosynthesis of 22-OH-MaR1 and 14-oxo-MaR1, resulting in an increased phagocytosis of *E. coli.* [102]. It should be noted that these data indicate that SPMs act at multiple levels and in multiple cells involved in CF pathophysiology in the airway.

#### 3.2.4. COVID-19

COVID-19, caused by severe acute respiratory syndrome coronavirus 2 (SARS-CoV-2)—a positive-sense single-stranded RNA virus—emerged in late 2019 as the causative agent of a global pandemic [103]. SARS-CoV-2 infection demonstrates broad systemic tropism, with particularly severe manifestations in the pulmonary system. Clinical presentations range from asymptomatic infection to life-threatening complications, including viral pneumonia, acute respiratory distress syndrome (ARDS), thrombotic coagulopathies, septic shock, and ultimately multi-organ dysfunction syndrome [104]. The outcome of severe COVID-19 cases is attributed to a deregulation of the immune response, leading to an exacerbated inflammatory state in which mainly the resolution phase of inflammation is impaired, in which the compounds released by alveolar macrophages and neutrophils in the initial phase of inflammation end up generating greater destruction, oxidative stress, and perpetuation of inflammation [105,106,107].

Since the debut of this ILD, one of the key points has focused on the search for efficient therapies against SARS-CoV-2, taking as a possible alternative the role of SPMs for their general effects in the resolution of acute inflammatory processes without major adverse effects that show other types of therapies, as in the case of immunosuppression [108,109]. Paradoxically, decreased levels of SPMs, in contrast to high levels of other pro-inflammatory mediators (such as prostaglandins), have been reported in several studies in patients with poor prognosis, suggesting that the activity of endogenous SPMs is insufficient against the cytokine storm produced in COVID-19 [110,111].

Therefore, a coadjuvant therapy has been proposed through the intake of omega-3 PUFAs since several studies have reported an increase in the levels of SPMs, possibly related to greater survival and control of the effects of COVID-19 in the short and long term [110,111]. In this regard, RvE1 and MaR1 inhibit leukocyte infiltration by down-regulating adhesion molecules in their membranes; it also reduces migration and infiltration of monocytes to the site of inflammation together with RvD1 [112]. In addition, it has been observed that RvD1 and RvD2 decrease the release of IL-8, monocyte chemotactic protein 1 (MCP-1), and macrophage inflammatory protein 1 (MIP-1α) in macrophages from individuals with CF stimulated with S1 protein from SARS-CoV-2. Likewise, some have been attributed to the ability to increase the secretion of anti-inflammatory cytokines such as IL-10 and TFG-β [113].

These SPMs, together with MaR1 and PD1, also act in the change of macrophage phenotype from M1 to M2, improving spherocytosis and phagocytosis of cellular debris, as well as reducing the secretion of pro-inflammatory cytokines and chemokines, similar to the actions observed in the case of LXA4 [114,115]. Another beneficial effect of SPMs is the action of RvD4, which, in addition to reducing PMN recruitment and infiltration, promotes the phagocytosis of blood clots and inhibits the release of neutrophil extracellular traps (NETs), promoting thrombus resolution characteristic of disseminated extravascular coagulation (DIC) in COVID-19 [116].

In the same vein, SPMs have demonstrated essential antiviral properties to prevent viral replication, as in the case of PD1, which has been shown to attenuate viral replication in mice infected with influenza virus [117]. Likewise, a recent study reports that supplementation with omega-3 PUFAs interferes with the binding of SARS-CoV-2 to the angiotensin II-converting enzyme receptor (ACE2), which is the receptor of choice for the binding of SARS-CoV-2 to the cell through the spike protein (ps) expressed on the surface of the envelope of this virus [118]. It is worth mentioning that the molecular mechanisms involved in the action of SPMs in long COVID have not been established, so further studies are needed concerning this clinical entity.

In contrast, SPMs demonstrate therapeutic potential across these molecular pathways, offering a dual COVID-19 resolution and prevention approach. Derived from PUFAs, these bioactive molecules, available through nutritional supplementation or pharmaceutical formulations, may emerge as critical components in future therapeutic strategies against SARS-CoV-2 and other virus infections. Their multimodal actions position SPMs as promising candidates for addressing the complex pathophysiology of COVID-19 [119,120].

### 3.3. Preclinical and Clinical Evidence of Pro-Resolving Lipid Mediators in the Management of Pulmonary Diseases

The development of novel therapeutic interventions for inflammatory lung diseases (ILDs) has emerged as a critical research priority. Over the past decade, numerous preclinical and clinical studies have investigated the therapeutic potential of SPMs in asthma, chronic obstructive pulmonary disease (COPD), and cystic fibrosis (CF) (Table 1). However, interpretation of these findings requires caution, as the cited clinical trials employed varying doses of PUFAs, precluding definitive conclusions or specific dosage recommendations at this stage.

#### 3.3.1. Asthma

Evidence suggests that SPMs are endogenously produced during airway inflammatory responses. Notably, reduced SPM levels have been mechanistically linked to asthma pathogenesis [127], highlighting their potential therapeutic and protective roles in this disease. Among SPMs, lipoxins (LXs) have been extensively investigated in asthma pathophysiology. Clinical studies reveal impaired biosynthesis of both LXA4 and LXB4 in severe asthma, with particularly strong correlations observed between decreased LXA4 levels in exhaled breath condensates and deteriorating pulmonary function [128,129,130,131]. Decreased levels of LXs have also been found in asthma exacerbations [132], exercise-induced asthma [133], and aspirin-intolerant asthma [134,135,136]. Levy et al. [137] demonstrated that LXA can inhibit airway hyperresponsiveness and inflammation. Furthermore, treatment with stable LXA4 analogues has been reported to reduce airway inflammation and hyperresponsiveness dose-dependent parameters [42]. Similarly, reports have established that LXB4 significantly decreases airway inflammation, mucus metaplasia, and hyperresponsiveness [138].

In addition, the RvD and RvE series have been reported to benefit airway inflammation [18,139,140]. Intravenous administration of RvD1 and AT-RvD1 in OVA-sensitized mice significantly decreased the development of an allergic response in the airway through eosinophils and pro-inflammatory mediator activation, as seen in a study by Rogerio et al. [141]. In a study by Kim et al. [142], RvD1 dampened IgE production in B cells from asthma patients. AT-RvD1 has also been used to enhance anti-inflammatory and pro-resolution effects in mononuclear cells in severe asthma patients’ plasma by reducing TNF-α and IL-10. These effects were associated with a decrease in NF-κB activation [55].

On the other hand, there is current evidence that RvE1 facilitates the inflammation resolution of the airway in clinical models of acute asthma exacerbation [140]. Aoki et al. also reported that the production of IgE, accumulation of inflammatory cells in the airways, airway hyperresponsiveness, mucus production, and Th2 cytokine production were decreased in a murine model of asthma after intraperitoneal administration of RvE1 [18]. Additionally, airway inflammatory cell accumulation, hyperresponsiveness, and mucus production were dampened during the resolution phase with intravenous administration of RvE1 [143].

PD1 has been used at the preclinical level in asthmatic subjects as well. Decreased PD1 synthesis in eosinophils from severe asthmatic patients was reported by Miyata et al. [127]. Levy et al. [144] also found that the levels of PD1 were significantly lower in exhaled breath condensates collected from murine models with asthma exacerbations. When 20 ng of PD1 were administered intravenously, airway eosinophils, T lymphocyte recruitment, airway mucus, pro-inflammatory mediators, and airway hyperresponsiveness were decreased. MaR1 has also been used in experimental studies. Contrary to other SPMs, MaR1 levels increase lung damage at first [145]. However, the administration of MaR1 can decrease the number of eosinophils in bronchoalveolar lavage and IgE, IL-5, TNF-α, and IL-13 levels, as shown in other studies [59,146].

At the clinical level, observational studies regarding ω-3 fatty acid diets among asthmatic adults have shown ambiguous results [147,148,149,150]. However, ω-3 fatty acid-rich diets have been associated with lower asthma prevalence, incidence [151], and airway hyperresponsiveness [152,153]. In a cross-sectional study of 642 subjects conducted by Shahieda et al. [154], higher EPA and DPA were significantly associated with decreased non-specific bronchial hyperresponsiveness (NSBH) risk. In addition, the total higher ω-3 levels were associated with decreased NSBH risk (OR = 0.92). In the Coronary Artery Risk Development in Young Adults (CARDIA) study, a recent prospective multicenter large cohort study, Li et al. [155] reported that ω-3 PUFA intake was inversely associated with the incidence of asthma, showing that its consumption has a protective effect. However, in randomized controlled trials, the evidence of ω-3 fatty acid consumption in asthmatic patients has been inconsistent [121,156,157,158,159,160,161,162,163,164].

In this sense, Thien et al. [165] performed a meta-analysis in which nine randomized controlled trials were included. The results showed no benefit or risk associated with dietary ω-3 fatty acids in people with asthma. Thus, given the current conflicting evidence, the European Academy of Allergy and Clinical Immunology recently released a statement highlighting no official recommendation for ω-3 PUFAs use in asthma—at least, not until more standardized multicentric randomized controlled trials have been performed [166]. On the other hand, in a recent randomized placebo-controlled double-masked trial, Mickleborough et al. [167] evaluated the beneficial effects of PCSO-524™ (Lyprinol^®^/Omega XL in asthmatic patients^®^), which is rich in ω-3 fatty acids. The study reported significantly reduced airway inflammation and bronchoconstriction following a dry gas airway challenge. These findings confirm those by Emelyanov et al. [163] and Wood et al. [168]; however, large-scale clinical studies using this drug in asthmatic patients are required.

Evidence suggests that asthma’s developmental origins are frequently established in early childhood [169,170], prompting investigations into preventive strategies during critical prenatal periods. Although clinical studies examining the immunomodulatory potential of maternal ω-3 fatty acid supplementation remain limited, emerging data reveal significant effects on offspring immune programming. These include transcriptional downregulation of Th2-associated cytokine genes in cord blood mononuclear cells and attenuation of neutrophil-mediated IL-13 and leukotriene B4 (LTB4) production, with both effects showing strong inverse correlations with ω-3 fatty acid incorporation in cellular membranes [171,172,173]. Furthermore, observational studies have suggested an association between an omega-3 PUFA-deficient diet during pregnancy and an increased risk of asthma in the offspring [174,175]. Numerous randomized, controlled omega 3 PUFA supplementation trials in pregnant women have been conducted [13,176,177,178,179,180,181,182,183,184]. Various clinical trials provided inconsistent evidence that increased ω-3 fatty acid intake in pregnancy reduces asthma in the offspring, finding poor or no association with asthma [181,185].

Nevertheless, the Copenhagen Prospective Studies on Asthma in Childhood 2010 (COPSAC 2010), a single-center, double-blind, placebo-controlled, parallel-group trial conducted by Bisgaard et al. [13], recently reported that daily supplementation of ω-3 fatty acid starting at 24 weeks of gestation leads to a 30.7% statistically significant relative reduction of risk of persistent wheeze or asthma in 3-year-old children (HR, 0.69; 95% CI, 0.49 to 0.97; *p* = 0.035). The higher doses of ω-3 fatty acid supplementation (2.4 g daily) and the evaluation of the outcome of overall wheeze rather than wheeze with sensitization may explain why these findings differ from the results of other authors [178,179,184]. Also, mothers with the lowest blood concentrations of EPA and DHA at the beginning achieved the greatest risk reduction, suggesting a patient’s subpopulation that may benefit the most from this protocol. However, the long-term safety of ω-3 fatty acid supplementation during pregnancy requires further randomized controlled studies.

#### 3.3.2. Chronic Obstructive Pulmonary Disease

The initial clinical evidence linking PUFAs to COPD outcomes emerged from the work of Sharp et al. [186], who identified a protective association between increased fish consumption (a primary source of ω-3 PUFAs) and attenuated decline in forced expiratory volume in one second (FEV1) among smokers. Subsequent epidemiological investigations have supported this observation, such as a large-scale study by Shahar et al. involving 8960 current and former smokers [187]. This work showed a significant inverse relationship between ω-3 PUFA intake and COPD severity while establishing that higher consumption of ω-3 PUFAs and fish products predicted better-preserved lung function in smoking populations [188].

Another population-based cohort study demonstrated that the percentage of energy from dietary fat, age, and plasma IL-6 were negative predictors of FEV1 and forced vital capacity (FVC) in men [189]. Furthermore, a cross-sectional study that analyzed the dietary habits related to PUFA consumption in smokers and lifetime non-smokers found significant differences in the dietary consumption of ω-3 PUFAs between these two groups and lower levels of DHA and EPA in smokers. Considering this, the researchers suggested that PUFAs might interfere with smoking habits, and the increase in ω-3 consumption may become a perspective in the prevention or treatment of smoking [122]. These findings are consistent with results reported in a cross-sectional study of 93 patients with COPD and 108 controls in Pakistan, where dietary intake of ω-3 PUFAs was lower in patients with COPD than in controls. Furthermore, PUFA intake levels decreased with disease severity [190]. Novgorodtseva et al. also studied the red cell membrane composition in patients with COPD, finding a decrease in the EPA percentage, an increase in the percentage of AA, and an increase in the AA/EPA ratio. This observation indicated an increased susceptibility of COPD patients to inflammatory eicosanoid production. This alteration in membrane composition was correlated to an increase in circulating inflammatory mediators, including TNF-α and TGF-β, in patients with COPD [123].

Conversely, Atlantis et al. [14] systematically reviewed two studies about supplementing specific PUFAs and their association with inflammation and functional capacity in COPD. Firstly, one study was an 8-week randomized controlled trial conducted in 80 COPD patients in the Netherlands that showed that PUFA supplementation significantly improved exercise capacity compared with the control group. Despite this, PUFA supplementation did not affect FEV1 or inflammation [191]. The second study was a cross-sectional investigation conducted on 250 COPD patients in Spain. It was reported that associations between specific dietary ω-3 fatty acids and inflammation decrease were inconsistent [192]. The authors concluded that evidence provides weak support for using ω-3 fatty acid supplementation to reduce chronic inflammation and some support for improving functional capacity in COPD patients [14].

In addition, two intervention trials with ω-3 PUFAs in COPD by Sugawara et al. [193,194] are aligned with its positive effect on COPD. The first is a randomized trial in which 32 patients with COPD received supplementation with a nutritional drink enriched with vitamin A and ω-3 PUFAs (0.6 g/day) combined with low-intensity exercise for 12 weeks. They were compared with a control group, with improved weight, fat-free mass, exercise capacity, quality of life, and inflammation reported in the ω-3 PUFAs/exercise group. In the second randomized controlled trial in 36 patients with COPD, Sugawara et al. investigated the effects of an oral nutritional supplement enriched with PUFAs (ω-6: ω-3 ¼ 2: 1, quantity not specified); vitamins A, C, and E; b-carotene; and minerals incorporated into a 12-week home-based pulmonary rehabilitation program. In the intervention group, positive effects were observed on fat mass, respiratory muscle strength, 6 min walk distance, and systemic inflammation (CRP, IL-6, TNF-α, and IL-8) compared to the control group taking the rehabilitation program without nutritional supplementation. Therefore, due to the design of these two studies, the specific effects of PUFAs could not be assessed because combined nutritional interventions were used.

A study by Lemoine et al., with secondary data from The National Health and Nutrition Examination Survey (NHANES), assessed the relationships between ALA or EPA + DHA intake and respiratory symptoms among US adults with COPD. The study included 878 participants, with a mean age of 60.6 years, of which 48% were current smokers. Logistic regression models, adjusting for age, gender, race, body mass index, FEV1, education, smoking status, pack-years, total caloric intake, and ω-6 (LA) intake, demonstrated no primary associations between ω-3 intake and respiratory symptoms. However, when interaction terms were used to determine potential modification of relationships by personal characteristics or exposures, it was observed that at lower levels of LA intake, increasing ALA intake was associated with reduced odds of chronic cough and wheezing [195]. A clinical trial is underway to evaluate the feasibility of a food voucher program and dietary counselling to increase the consumption of ω-3 fatty acids in individuals with COPD [196].

Exposure to cigarette smoke (CS) induces alterations in the lungs’ endothelial cells, particularly endothelial activation and breaches in its barrier function, which may facilitate leukocyte and minor plasma extravasation in the lung parenchyma, contributing to chronic inflammation [197]. Interestingly, smoking also was demonstrated to inhibit surface expression of CFTR protein, preventing its ability to function as a chloride channel, suggesting that aberrant levels of AA/EPA plus DHA ratios may also result from the impairment of CFTR function in smoke-induced COPD [198,199]. Other studies have shown that reduced CFTR expression in alveolar macrophages or CFTR deficiency in epithelial cells confers a proinflammatory phenotype and induces apoptosis [200]. In this sense, Kaza et al. found that after cigarette smoke exposure, ferrets exhibited CFTR dysfunction, increased mucus viscosity, delayed mucociliary clearance, airway wall thickening, and airway epithelial hypertrophy [201].

The mechanisms behind CFTR dysfunction promoting epithelial damage are currently unclear. It has been suggested that CFTR dysfunction contributes to emphysema by regulating proinflammatory ceramide signaling [200]. In addition, CFTR inhibition has been shown to increase the permeability of the pulmonary vasculature, and it has been hypothesized that this may lead to increased trafficking of inflammatory cells to the lungs [202]. It has also revealed that CFTR inhibition leads to disrupted distribution of β-catenin at the endothelial intercellular junction and rearrangement of actin. These changes have been linked to impaired barrier function and increased vascular permeability, which could further exacerbate the inflammatory response and contribute to the development of chronic lung diseases. These findings underscore the importance of maintaining a healthy endothelial barrier in the lungs and suggest that targeting CFTR may be a promising strategy for preventing or treating smoke-induced lung damage [202].

#### 3.3.3. Cystic Fibrosis

One of the well-documented metabolic defects in CF is PUFA imbalance level, including the increase in AA levels and the decrease in DHA and EPA levels [203,204,205,206,207,208,209,210]. AA is a substrate not only for cyclooxygenases [211] but also for lipoxygenases (5-LOX, 12-LOX, and 15-LOX), which subsequently convert into leukotrienes, lipoxins, and hepoxilins [212]. Therefore, it is unsurprising that abnormal levels of 15-LOX, LXA4, and RvD1 concentrations have been reported in CF patients [100,213,214,215].

Numerous studies have shown that decreased SPMs derived from DHA concentrations may contribute to CF’s pathobiology, and using various SPMs may have a protective effect. Furthermore, RvD1, RvD2, RvD3, and MaR1 have been proven capable of reducing mucus metaplasia, parenchymal inflammation, leukocyte infiltration, and polymicrobial sepsis severity, and enhancing E. coli phagocytosis by macrophages at the preclinical level [99,102,216].

Randomized controlled trials have attempted to assess whether SPM intake may benefit CF patients [15,124,217,218,219,220,221], and some findings confirmed potential therapeutic effects, while others were inconclusive. In this sense, Watson et al. [221] conducted a meta-analysis of five randomized clinical trials evaluating the evidence regarding the beneficial effects of omega-3 fatty acid supplementation in CF patients. They reported that ω-3 fatty acid intake may provide therapeutic benefits for people with CF with relatively few adverse effects. Likewise, Lawrence et al. [124] performed a six-week randomized controlled trial that reported an improvement in lung function (*p* = 0.006) in CF patients treated with ω-3 fatty acids, although other authors reported that the intake of PUFAs did not produce any differences in lung function [15,217,219]. Additionally, Hanssens et al. [217] compared omega-3 fatty acid supplementation with a placebo control group after 1 year of treatment in CF patients, finding a significantly decreased number of pulmonary exacerbations (*p* < 0.01). However, the total available data in the literature is very limited in promoting the systematic use of PUFAs in CF; hence, larger randomized controlled trials are required to determine further therapeutic long-term effects.

Moreover, drugs targeting SPM biosynthesis have been studied in clinical trials in CF patients [222,223,224,225,226,227]. Lenabasum (JBT-101), a selective cannabinoid receptor type 2 agonist, triggers the resolution of inflammation by increasing the biosynthesis of LXA4. In a double-masked, placebo-controlled phase 2 trial, a trend toward a reduced risk of pulmonary exacerbations was reported, as well as a significant decrease in IL-8, neutrophil, elastase, and IgG [227]. A larger phase II trial on lenabasum is underway [228,229]. Similarly, acebilustat (CTX-4430), an oral inhibitor of LTA4 hydrolase that increases LXA4 biosynthesis by shutting down LTB4, has been reported to also generate a significant decrease in PMN numbers and neutrophil elastase levels in the sputum of CF patients [222].

#### 3.3.4. COVID-19

Recent research has prioritized the development of therapeutic interventions against SARS-CoV-2 infection, with growing evidence supporting specialized pro-resolving mediator (SPM) administration in COVID-19. Preclinical studies demonstrate that SPM deficiency correlates with disease severity and that these molecules can limit extrapulmonary viral dissemination, a phenomenon also observed with other respiratory viruses like H1N1 influenza. These findings highlight SPMs as promising candidates for modulating the dysregulated immune response in severe COVID-19 [230]. In this regard, a study by Morita et al. showed how PDs can inhibit influenza virus replication and that their absence is associated with a higher degree of virulence and pathogenicity of the virus [117]. Thus, it is likely that positive modulation of SPMs contributes to counteracting the cytokine storm, inflammation, and cellular infiltrate characteristic of COVID-19 [231]. The potential role of specialized pro-resolving mediators (SPMs) in long COVID pathogenesis represents a critical area for investigation, though current evidence remains limited. Preliminary data suggest that SPMs may modulate persistent inflammatory pathways characteristic of post-acute sequelae, but rigorous clinical studies are needed to establish causal mechanisms and therapeutic potential.

Under this premise, clinical trials have reported promising results regarding omega-3 PUFA administration in COVID-19 patients. A study evaluating the association between the use of different dietary supplements in 445850 individuals showed that omega-3 PUFA consumption was associated with a lower probability of having a positive test for COVID-19, highlighting a possible protective and preventive effect against the disease [232]. Equally, a study showed that patients with COVID-19 had a lower omega-3 PUFA index and an inverse association between this index and the need for mechanical ventilation (OR: 0.459) and death (OR: 0.28) in severe COVID-19, highlighting the importance of PUFA supplementation in these patients [233]. Similar results were reported in a systematic review of 18 clinical epidemiologic studies [126].

In this vein, a randomized, double-masked clinical trial in 128 critically ill patients infected with SARS-CoV-2 virus by Doaei et al. showed that supplementation with 1000 mg omega-3 for 14 days was associated with a significantly higher 1-month survival rate, higher levels of arterial pH and bicarbonate (HCO3), and lower levels of creatinine, blood urea nitrogen, and potassium compared to the control group (*p* < 0.05) [125]; such findings may indicate a lesser degree of viral renal and respiratory involvement in critically ill patients. Interestingly, a case report of an elderly patient infected with SARS-CoV-2 who was treated with isosapent ethyl (IPE), an omega-3 derivative, showed a faster resolution of disease symptoms compared to other patients [234]; however, it is important not to generalize such results, as the role of IPE needs to be proven in a larger population, taking into account the inter-individual variability observed in patients with COVID-19. Nevertheless, it is appropriate to continue studying the relationship between these dietary supplements and COVID-19; there are currently phase III clinical trials aimed at evaluating the therapeutic role of omega-3 PUFAs in patients with COVID-19 and/or long COVID-19 [235,236,237].

## 4. Conclusions

In recent decades, pulmonology has witnessed significant therapeutic advancements for ILDs. While the molecular immunology of asthma, COPD, cystic fibrosis, and COVID-19 remains an active research frontier, novel pharmacotherapeutic strategies have emerged targeting the dual pro-resolution and anti-inflammatory properties of specialized pro-resolving mediators (SPMs) from PUFAs. Substantial evidence now implicates SPM deficiency in the pathogenesis of these conditions, driving translational research into their therapeutic potential as a novel class of ILD treatments. Hence, several preclinical and clinical studies have been conducted to evaluate their impact, which has led to promising results in asthma prevention in offspring and the use of various drugs targeting the biosynthesis of SPMs in CF, which are currently undergoing phase 2 of clinical trials. Research in PUFA intake and their homologs may lead to positive results in the future. Therefore, more randomized, multicentered, controlled trials with larger samples and longer follow-ups are required to clarify its true impact in clinical practice.

## Figures and Tables

**Table 1:** Summary of critical clinical evidence regarding specialized pro-resolving lipid mediators and pain-related conditions.

Authors	ILD	Methodology	Results
Thien et al. [121]	Asthma	A meta-analysis evaluating high marine ω-3 fatty acid diet in asthma.	No beneficial effect from ω-3 fatty acid intake was observed on FEV1, peak flow rate, asthma symptoms, asthma medication use, or bronchial hyperreactivity.
Bisgaard et al. [13]	Asthma	A randomized, double-blind, placebo-controlled study assessed 2.4 g of ω-3 fatty acid intake on the risk of asthma in the offspring of 736 pregnant women.	There was a 30.7% statistically significant relative reduction of risk of persistent wheezing for asthma in the offspring (HR, 0.69; 95% CI, 0.49 to 0.97; *p* = 0.035).
Broekhuizen et al. [122]	COPD	A randomized, double-masked, placebo-controlled, 8-week study evaluated the effects of PUFA treatment in 80 COPD patients.	PUFA supplementation significantly improved exercise capacity (MD 9.7 W; 95% CI 2.5 to 17.0; *p* = 0.009) but did not affect FEV1 or inflammation.
Sugawara et al. [123]	COPD	EPA and DPA supplementation on lung function in COPD patients.	Supplementation of EPA (*p* = 0.006) and DPA (*p* = 0.022) was significantly associated with a slower FEV1 decline.
Watson et al. [124]	CF	A meta-analysis of five randomized controlled trials was conducted to evaluate the benefit of omega-3 fatty acids in CF.	One study reported reduced pulmonary exacerbations and antibiotic use when the subjects were treated with PUFAs. In another six-week study, sputum levels were reduced, and lung function and clinical status improved when taking omega-3 supplements.
Recchiuti et al. [98]	CF	A randomized, double-masked, placebo-controlled, phase-1 study that evaluated the effects of acebilustat (CTX-4430) in CF patients.	The treated group had a 58% and 65% reduction in elastase and sputum neutrophil counts, respectively (*p* < 0.05).
Doaei et al. [125]	COVID-19	A randomized, double-masked clinical trial study evaluated the effects of omega-3 supplementation on 128 critically ill patients infected with COVID-19.	Patients with COVID-19 who were critically ill showed improved respiratory and renal function, leading to 1-month-higher survival rates (*p* < 0.05).
Mazidimoradi et al. [126]	COVID-19	A systematic review evaluating PUFA administration on COVID-19.	Omega-3 supplements reduced the risk of COVID-19 by 12–21%. On the other hand, a deficiency in omega-3 has been linked to severe COVID-19 symptoms, a higher need for mechanical ventilation, hospitalization, and increased mortality.

Abbreviations: ILD: inflammatory lung disease; COPD: chronic obstructive pulmonary disease; CF: cystic fibrosis; ω-3: omega-3; EPA: eicosapentaenoic acid; DPA: docosapentaenoic acid; PUFAs: polyunsaturated fatty acids; FEV1: forced expiratory volume in one second.

## Data Availability

No new data were created or analyzed in this study. Data sharing is not applicable to this article.

## Abbreviations

The following abbreviations are used in this manuscript:

Abbreviation	Definition
ASL	Mucociliary clearing
CF	Cystic fibrosis
COX-2	Cyclooxygenase 2
COPD	Chronic obstructive pulmonary disease
CYPs	Cytochromes P450
DHA	Docosahexaenoic acid
DPA	Docosapentaenoic acid
EPA	Eicosapentaenoic acid
FEV1	Forced expiratory volume in one second
ILD	Inflammatory lung diseases
LOX	Lipoxygenases
LXA4	Lipoxins A4
mar-01	Maresin 1
NSAID	Non-steroidal anti-inflammatory drug
NO	Nitric oxide
NPD1	Neuroprotectin 1
OS	Oxidative stress
PD1	Protectin 1
PMN	Polymorphonuclear neutrophil
PUFAs	Polyunsaturated fatty acids
RvD1	Resolvin D1
RvD2	Resolvin D2
RvD3	Resolvin D3
RvD4	Resolvin D4
RvD5	Resolvin D5
RvD6	Resolvin D6
RvE1	Resolvin E1
RvE2	Resolvin E2
RvE3	Resolvin E3
ω-3	Omega-3
ω-6	Omega-6
